# Hypervalent iodine/TEMPO-mediated oxidation in flow systems: a fast and efficient protocol for alcohol oxidation

**DOI:** 10.3762/bjoc.9.162

**Published:** 2013-07-17

**Authors:** Nida Ambreen, Ravi Kumar, Thomas Wirth

**Affiliations:** 1 Cardiff University, School of Chemistry, Park Place, Cardiff CF10 3AT, UK

**Keywords:** alcohols, carbonyl compounds, flow chemistry, microreactor, oxidation

## Abstract

Hypervalent iodine(III)/TEMPO-mediated oxidation of various aliphatic, aromatic and allylic alcohols to their corresponding carbonyl compounds was successfully achieved by using microreactor technology. This method can be used as an alternative for the oxidation of various alcohols achieving excellent yields and selectivities in significantly shortened reaction times.

## Introduction

Oxidation of alcohols to carbonyl compounds plays an important role in organic chemistry. The transformation is traditionally achieved by using chromium-based reagents such as the Collins reagent, activated manganese dioxide, or procedures known as the Swern [1], Pfitzner–Moffatt [2] or Parikh–Doering oxidation [3]. In synthetic chemistry, selective methods for the oxidation of alcohols are highly sought after, and methods with the ability to differentiate between various functional groups are desired. The use of hypervalent iodine reagents in organic chemistry has increased during recent years [4–6]. Hypervalent iodine compounds in general have emerged as versatile oxidizing agents with compounds such as DMP (Dess–Martin periodinane) and IBX finding regular utility as highly selective oxidizing agents [7–9]. The use of the nitroxyl radical TEMPO (2,2,6,6-tetramethylpiperidine-1-oxyl) as a catalyst in the oxidation of alcohols has gained much attention in recent years [10–12]. The redox cycle involves beside TEMPO also the corresponding hydroxylamine and the oxoammonium cation, which oxidizes the alcohol and is converted to TEMPO–H [13]. Hypervalent iodine(III) reagents in combination with a catalytic amount of TEMPO have already been reported in highly selective oxidations of alcohols to carbonyl compounds [14].

The development of efficient flow-reactor systems for molecular transformations is an important area in organic synthesis. The introduction of more general platforms to perform reactions under continuous flow rather than in batch mode has led to improvements regarding safety and sustainability. Microreactor technology can be beneficial over classical approaches in a variety of chemical reactions. Many reactions can benefit from the properties of microreactors. Enhanced mass- and heat transfer and short diffusion distances can lead to better yields within shorter reaction times [15]. Herein, we describe the development of continuous-flow systems using hypervalent iodine reagents in the TEMPO-mediated oxidation of alcohols with the advantage of significantly shortened reaction times. Several other oxidative processes have already been reported in flow chemistry [16].

## Results and Discussion

**Figure 1:**
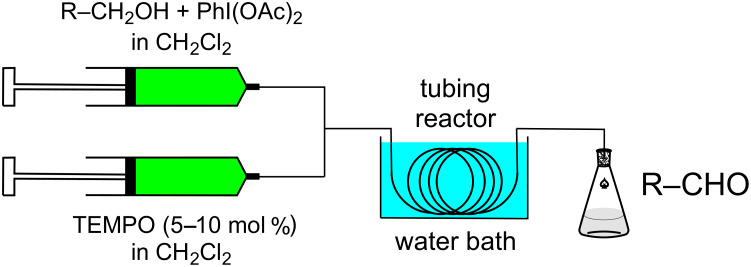
Flow setup for alcohol oxidations.

Benzyl alcohol was chosen as a substrate in order to examine the efficiency of the reaction and the microreactor flow system. In a batch reaction, the mixture of benzyl alcohol (**1a**) and (diacetoxyiodo)benzene (**2**) did not show any reaction after stirring for 12 h in dichloromethane at 35 °C. The addition of a catalytic amount TEMPO to the reaction mixture led to a rapid conversion to benzaldehyde (**3a**). For initial investigations of a flow system, a simple setup consisting of two syringes driven by a syringe pump, a T-connector and a tubing reactor (PTFE tubing, length: 4 m, internal diameter: 0.75 mm) was used for the oxidation of benzyl alcohol to benzaldehyde. The tubing reactor was inserted in a water bath at constant temperature as shown in Figure 1. In all flow experiments, the alcohol substrate **1** and oxidant **2** were mixed in one syringe, and the reaction started by combining this mixture with the solvent stream of a second syringe containing the catalyst TEMPO.

The reactions have been performed at 35 °C. With a residence time of 30 s the conversion to benzaldehyde was determined by GC to be 52%. Increasing the residence time to 1 min, 2 min and 4.5 min led to conversions of 65%, 79% and 95%, respectively. The reaction is incomplete if performed with amounts below 5 mol % of TEMPO (1 mol % TEMPO: 67% conversion, 2 mol % TEMPO: 81% conversion). Therefore all experiments were performed with at least 5 mol % TEMPO catalyst.

To extend the substrate scope, various benzylic, aliphatic and allylic alcohols were investigated. Good yields at short reaction times and a high selectivity towards the oxidized products were observed as shown in Table 1. Over-oxidation to the corresponding carboxylic acids was not detected and high selectivities were obtained.

**Table 1:** Products and yields in the oxidation of alcohols performed in a tubing reactor at a total flow rate of 0.4 mL/min (residence time: 4.5 min) at 35 °C.

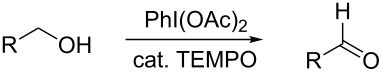					

Entry	Alcohol	Product	Reference^a^	Conversion (%)^b^	Yield (%)^c^

1	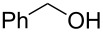	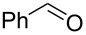	[17]	100	49
2	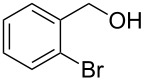	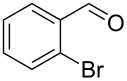	[18]	99	79
3	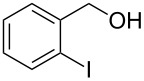	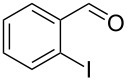	[19]	100	95
4	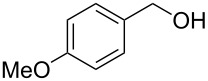	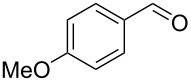	[17]	99	82
5	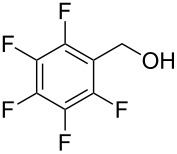	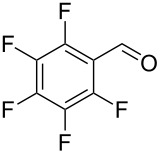	[20]	96	51
6	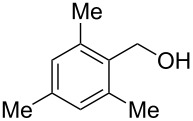	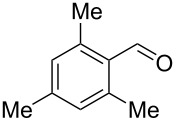	[21]	97	75
7	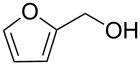	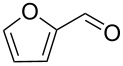	[17]	80	48
8	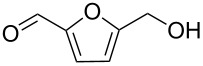	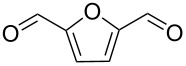	[22]	89	61
9	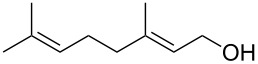	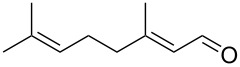	[17]	96	76
10	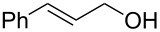	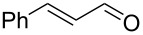	[17]	97	97
11	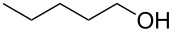	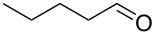	[23]	89	87
12	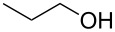	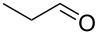	[24]	100	–
13	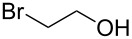	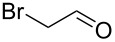	[25]	56	–
14	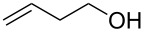	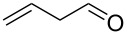	[26]	87	–
15	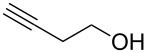	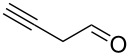	[27]	100	–
16	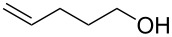	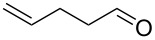	[28]	87	–
17	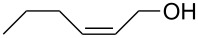	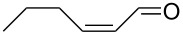	[19]	88	–
18	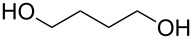	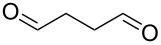	[29]	91	–
19	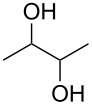	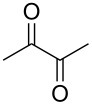	[30]	77	–
20	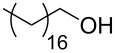	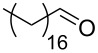	[31]	–	95
21	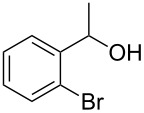	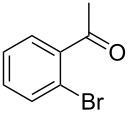	[32]	100	–
22	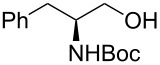	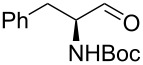	[33]	n.d.	52
23	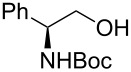	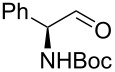	[33]	n.d.	40

^a^Spectral properties consistent with literature data. ^b^Conversions determined by GC. n.d.: not determined. ^c^Isolated yields.

**Scheme 1:**
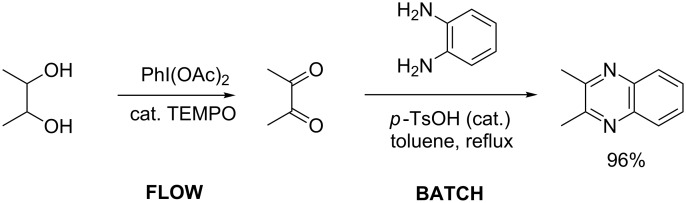
Oxidation–condensation sequence in the synthesis of 2,3-dimethylquinoxaline.

In larger scale reactions (0.5 g), the conversion monitored by GC was nearly quantitative. Isolated yields were lower than those indicated by GC and largely reflect losses from isolation procedures for individual products. The optimized reaction time of 4.5 min was then used to convert also a larger amount of benzyl alcohol (15 mmol) to benzaldehyde, which was isolated as the reaction product in 49% yield. These results provide further evidence that this flow process is effective for oxidation methods initially discovered and developed under batch conditions. The substrate scope of the catalytic method in this flow process reflects that of the method originally developed in batch. *N*-Boc protected (*S*)-phenylalaninol [34] (Table 1, entry 22) was oxidized to the corresponding aldehyde [35] without loss of optical purity as determined by the optical rotations of starting material and product [36]. *N*-Boc protected (*S*)-phenylglycinol [37] (Table 1, entry 23) suffered from some optical degradation probably during the work-up [38]. Aldehydes are rarely target molecules of pharmaceutical synthesis. These functional groups rather represent highly useful intermediates for subsequent reactions. The addition of 1,2-diaminobenzene to the crude oxidation product of 2,3-butanediol (Table 1, entry 19) allowed the direct and almost quantitative synthesis of 2,3-dimethylquinoxaline with *para*-toluenesulfonic acid as catalyst in the subsequent condensation reaction performed in a batch system (Scheme 1) [39] The oxidation–condensation sequence described here generates almost no byproducts except iodobenzene which can be removed very easily during the chromatographic purification of the product and should enable direct progression to the subsequent synthetic steps, without the need for isolation or purification of the intermediate aldehyde or ketone.

## Conclusion

In conclusion, a highly efficient and selective continuous-flow reaction for the oxidation of different alcohols was developed. Apart from short reaction times, high conversions and excellent selectivities were obtained. These features, together with the low toxicity of the reagents, make the process attractive compared to the batch reaction. The economical and benign oxidation is broadly applicable to a wide range of alcohols.

## Experimental

**General: **^1^H NMR and ^13^C NMR spectra were recorded on a AV-400 Bruker spectrometer by using the solvents indicated with 400 and 100 MHz, respectively. All reactions were monitored by thin-layer chromatography that was performed on precoated sheets of silica gel 60. GC analyses were performed on a GC-FID (Varian 3900) chromatograph. All purchased chemicals were used without further purification.

### General procedure for the alcohol oxidation in flow

Solutions of (diacetoxyiodo)benzene (1.1 equiv) and the alcohol (50 mg) in CH_2_Cl_2_ (1.5 mL) and 2,2,6,6-tetramethyl-1-piperidinyloxyl (TEMPO) (5–10 mg, 10–20 mol %) in CH_2_Cl_2_ (1.5 mL) were loaded in two syringes. Both syringes were placed in a syringe pump (Fusion 100) and connected via a T-piece to a tubing reactor (PTFE, length: 4 m, internal diameter: 0.75 mm). The tubing reactor was immersed in a thermocontrolled water bath at 35 °C. The total flow rate was adjusted to 0.4 mL min^–1^ resulting in a residence time of 4.5 min. The reaction mixture exiting the flow reactor was quenched with water and, after completion of the reaction, extracted with CH_2_Cl_2_. The combined organic layers were dried over magnesium sulfate and the solvents were removed in vacuo. Direct analysis with GC allowed the determination of the conversion by comparison of the product peak with the peak of the starting alcohol.

### Large scale oxidation of benzyl alcohol in flow

The reaction was performed with the Vapourtec E-Series using a PFA tubing reactor. Benzyl alcohol (2 g, 18.5 mmol) and (diacetoxyiodo)benzene (6.3 g 19.5 mmol) were dissolved in CH_2_Cl_2_ (120 mL). 2,2,6,6-Tetramethyl-1-piperidinyloxyl (TEMPO) (160 mg, 1 mmol) was dissolved in CH_2_Cl_2_ (120 mL). Both solutions (flow rates: 2 mL min^–1^ each) were mixed in a T-piece before entering the tubing reactor (volume: 10 mL) resulting in a residence time of 5 min. After constant flow had been achieved, 200 mL of the reaction solution was collected in a flask containing water (20 mL) as a quenching agent. After completion of the reaction, the organic phase was removed and the aqueous phase was extracted with CH_2_Cl_2_ (3 × 20 mL). The combined organic layers were dried over magnesium sulfate, and the solvents were removed in vacuo. The crude reaction mixture was purified by flash chromatography on silica using hexane/ethyl acetate (9:1) as eluent. Some benzaldehyde was lost during the drying processes and 0.8 g (7.5 mmol, 49%) was isolated.

### Procedure for the aldehyde and diamine condensation

1,2-Phenylenediamine (0.59 mg 0.55 mmol) was added to the crude oxidized product of 2,3-butanediol (50 mg 0.55 mmol) and dissolved in toluene in a round-bottom flask, and *p*-toluenesulfonic acid was added as catalyst. The reaction mixture was heated under reflux for 2 hours and monitored by TLC. After the completion of the reaction the solvent was evaporated, and the reaction mixture was extracted with CH_2_Cl_2_ and water. The organic layers were dried over magnesium sulfate and the solvents were removed in vacuo. The ^1^H NMR analysis showed a clean spectrum of the condensation product.
